# An Overview of Gaucher Disease

**DOI:** 10.3390/diagnostics14242840

**Published:** 2024-12-17

**Authors:** Daniela Anahí Méndez-Cobián, Sandra Guzmán-Silahua, Diana García-Hernández, Julian Conde-Sánchez, Yaocihuatl Castañeda-Borrayo, Kylee Louise Duey, Maria G. Zavala-Cerna, Benjamín Rubio-Jurado, Arnulfo Hernán Nava-Zavala

**Affiliations:** 1 Unidad de Investigación Epidemiológica y en Servicios de Salud, Centro Médico Nacional de Occidente Órgano de Operación Administrativa Desconcentrada Jalisco, Instituto Mexicano del Seguro Social, Guadalajara 44329, Jalisco, Mexico; daniela_anahi17@outlook.com (D.A.M.-C.); guzmansilahuasandra@gmail.com (S.G.-S.); diana_salmaq@hotmail.com (D.G.-H.); julian.conde7874@alumnos.udg.mx (J.C.-S.); dra_yao@hotmail.com (Y.C.-B.); 2 Programa de Médico Pasante en Servicio Social SSJ, Universidad de Guadalajara Centro Universitario del Sur., Ciudad Guzmán 49000, Jalisco, Mexico; 3 School of Medicine International Program, Universidad Autónoma de Guadalajara, Av. Patria 1201, Zapopan 45129, Jalisco, Mexico; kylee.duey@edu.uag.mx; 4 Immunology Research Laboratory, Decanato Medicina, Universidad Autónoma de Guadalajara, Zapopan 45129, Jalisco, Mexico; maria.cerna@edu.uag.mx; 5 Programa de Médico Pasante en Servicio Social en Investigación, Dirección General de Calidad y Educación en Salud, Secretaría de Salud, Guadalajara 44329, Jalisco, Mexico; 6 Servicio de Salud en el Trabajo, Unidad de Medicina Familiar #53, OOAD Jalisco, Instituto Mexicano del Seguro Social, Zapopan 45170, Jalisco, Mexico; 7 Departamento Clínico de Hematología, División de Onco-Hematología, UMAE Hospital de Especialidades, Centro Médico Nacional de Occidente, Instituto Mexicano del Seguro Social, Guadalajara 44329, Jalisco, Mexico; 8 Departamento de Inmunología y Reumatología del Hospital General de Occidente, Secretaria de Salud Jalisco, Zapopan 45170, Jalisco, Mexico

**Keywords:** Gaucher disease, hereditary disease, lysosomal storage diseases, treatment

## Abstract

Background: Gaucher disease (GD) is a rare autosomal recessive disorder caused by mutations in the GBA1 gene that lead to a deficiency in the glucocerebrosidase gene. This deficiency results in the accumulation of glucocerebrosides in macrophages, primarily affecting the liver, spleen, and bone marrow. Focusing on the Mexican population, this study aims to review GD’s epidemiology, clinical manifestations, and treatment options to enhance early diagnosis and optimize treatment outcomes. Methods: This study is a comprehensive literature review analyzing epidemiological data, clinical presentations, and current therapeutic approaches for Gaucher disease, including enzyme replacement therapy (ERT) and substrate reduction therapy (SRT). Conclusions: Early diagnosis and individualized treatment, primarily through enzyme replacement therapy, significantly improve the prognosis of patients with Gaucher disease, particularly type 1. Continued research is required to enhance therapeutic approaches for the neuropathic types and better understand the disease’s epidemiology in Mexico. These efforts will contribute to improved clinical outcomes and quality of life for patients.

## 1. Introduction

Gaucher disease (GD) was first described by the French physician Philippe Charles Gaucher in 1882. During the preparation of his doctoral thesis, Gaucher performed the autopsy of a 32-year-old patient who had died after presenting a prominent splenomegaly, describing it as “Idiopathic Hypertrophy of the Spleen without Leukemia” [1]. Later, in a similar case reported in 1885, the disease was named Gaucher disease [2]. In 1904, Brill suggested that the disease was hereditary and demonstrated that it affected the liver, lymph nodes, and bones [2].

In 1920, the first patients with neurological manifestations were described [2]. In 1934, Aghion demonstrated that Gaucher cells infiltrate the reticuloendothelial organs; this histological finding was considered pathognomonic and showed an abnormal accumulation of glucocerebrosides [2,3]. In 1960, Brady and others demonstrated the biochemical basis of lipid storage in GD, showing that the biosynthetic pathways of glucocerebroside were normal in affected patients but that the primary degradation pathway of glucocerebrosidase was defective due to a deficiency or lack of lysosomal hydrolase β-glucocerebrosidase (GCase) activity, which is the enzyme responsible for the intracellular hydrolysis of glucosylceramide and other related sphingolipids, leading to the accumulation of these substrates in macrophage–monocyte system cells [2,4,5].

Over time, GD was characterized as an autosomal recessive hereditary disorder, with the underlying anomaly located on chromosome 1 (1q2.1) [3]. To date, 279 variants of GD have been reported in the Leiden Open Variation Database and more than 400 variants have been registered in the Human Gene Mutation Database (HGMD) [5]. The alterations appear throughout the GBA gene, with the most common being due to nucleotide changes. Moreover, approximately 20% of GD alterations are deletions, insertions, and complex rearrangements [6].

## 2. Definition

Gaucher disease is a rare, genetic, hereditary disease in which mutations occur in the GBA1 gene, which codifies the GCase enzyme [7]. The disease is distinguished by lysosomal storage in macrophages (Gaucher cells) in the liver, spleen, bones, and bone marrow. Due to the organs involved and the different degrees of severity that may manifest, heterogeneous genotypic and phenotypic patterns are shown [7].

## 3. Epidemiology

Lysosomal storage diseases (LSDs) involve more than 70 entities; they are individually rare but collectively affect 1 in every 5000 live births, with no ethnic preference [3].

Gaucher disease is the most prevalent LSD [5], with an incidence of 1 case per 40,000–60,000 births in the general population; however, it can occur in 1–800 births within the Ashkenazi Jewish population [8]. For Ashkenazi Jewish people, GD may not be considered rare; however, epidemiological data for Gaucher disease in one ethnicity may not represent other ethnicities [9]. A meta-analysis by Wanh Meimei in 2023 reported a global prevalence of GD at 1.5 cases per 100,000 live births [9].

The first records of Gaucher disease in Mexico were from 1966 at the Centro Médico Nacional “La Raza” of the Instituto Mexicano del Seguro Social (IMSS) [6]. Due to increased cases, the first multidisciplinary team to manage GD arose in 1991. By 2005, a general census identified 68 cases residing in the states of Sinaloa, Nuevo León, Michoacán, Morelos, Jalisco, Puebla, Baja California Sur, Coahuila, and Mexico City [6]. In a cohort from 2021 including GD cases from México and Spain, type 1 GD was reported to be more common among the Mexican population [5]. This disease is among the 20 rare diseases recognized in Mexico [10].

## 4. Classification and Clinical Manifestations

There are three types of GD patients according to the presence and rapid progression of neurological manifestations [5,11]. The three GD types can be described as the following (Table 1):

- Type 1 (GD1) is the most common and has been traditionally defined as non-neuropathic because it was known that there is the absence of primary central nervous system disease [12]; however, in recent years, we have learned that there is neurological involvement [13]. GD1 is characterized by hepatosplenomegaly, pancytopenia, and skeletal involvement presence, without neurological manifestations, with a variable age for the onset of symptoms [5].
- Type 2 (GD2) is the acute neuropathic type, which displays severe hepatosplenomegaly and progressive neurological deterioration [5]. GD2 is the phenotype with the worst prognosis; it manifests before 6 months of age, and a few cases may present perinatally with congenital ichthyosis or hydrops fetalis [14]. GD2 patients usually die in infancy or early childhood despite multiple interventions [15].
- Type 3 (GD3) is also known as chronic neuropathic GD and is characterized by variable visceral damage [5]. GD3 has neurological implications, especially oculomotor movements that usually appear in early childhood with slower progression [14].

This summary classification provides a general clinical parameter that is helpful because lysosomal storage diseases exhibit a comprehensive and complicated spectrum of phenotypes [6].

**Table 1:** Gaucher disease classification.

	Type 1	Type 2	Type 3
Prevalence	90–95%	<5%	<5%
Presentation age	Childhood/adulthood	Infancy	Childhood
Survival (years)	Ages 6–80	Ages 2–3	Ages 30–40
Clinical presentation	Type 1 is variable, ranging from asymptomatic throughout life to early-onset forms in childhood. The clinical presentation includes the following: Fatigue; Growth retardation and delayed puberty; Hepatosplenomegaly; Mucocutaneous bleeding; Spontaneous hematomas; Anemia; Painful bone crises, mainly in the pelvis and lower limbs; Erlenmeyer flask bone deformity; Pulmonary fibrosis; Proteinuria and hematuria (uncommon).	Type 2 is characterized by several early and acute neurological deteriorations that start in 3–6-month-old babies. The clinical presentation includes the following: Growth retardation; Hydrops fetalis; Congenital ichthyosis; Hepatosplenomegaly; Bone pain. The characteristic triad is the following: Opisthotonus; Bulbar signs (severe swallowing disorders); Oculomotor paralysis (bilateral fixed strabismus).	Type 3 is similar to type 2 but with a slower clinical presentation (chronic). The clinical presentation includes the following: Growth retardation; Strabismus; Gaze palsy; Progressive dementia; Myoclonus; Corneal opacity; Seizures.

Modified from [11]. With actualizations from [2,8,12,16].

### 4.1. Manifestations in the Skeletal System

Musculoskeletal conditions can also affect patients’ quality of life. These include bone marrow infiltration by Gaucher cells, microvascular occlusion in the bone, bone infarction, recurrent avascular osteonecrosis, cortical thinning, and impaired bone remodeling, leading to osteolytic lesions and bone density loss [17,18]. Various mechanisms affect the bones, such as bone pain, increased fracture susceptibility, joint damage with secondary osteoarthritis, bone deformity, and disability, sometimes necessitating orthopedic surgeries [17,18].

Studies have shown that up to 90% of Gaucher disease patients experience bone involvement, as evidenced by imaging studies such as X-rays. The most significant finding is the appearance of the Erlenmeyer flask deformity (caused by impaired bone remodeling and elongation of the metaphyseal region of the long bones), with the femur being one of the most affected bones [17].

### 4.2. Novelties in Gaucher Disease Clinical Classification

Close, long-term clinical monitoring is often necessary to determine the specific GD type [15]. Over the years, the distinction among the three GD phenotypes as a consequence of superposition manifestations and the existence of intermediate phenotypes has been analyzed. Neurological involvement in patients with GD1, such as parkinsonism, peripheral neuropathy, and nerve root compressions, has also been described [13].

GD types 2 and 3 feature primary central nervous system (CNS) disease; therefore, it is essential to establish the differences between GD types to provide appropriate treatment and care to patients, which varies significantly between GD2 and GD3 [15].

The new significant clinical differences between GD types are in the eye findings: in GD2, horizontal and vertical slowed or absent saccades, and in GD3, slowed horizontal saccades. In neurological findings, significant clinical differences include the following: in GD2, hyper- or hypotonicity, seizures, gross motor developmental delay, and loss of developmental milestones, and in GD3, gradual myoclonic epilepsy, learning disorders, intellectual impairment, and hydrocephalus [15].

Type 3 GD has a heterogeneous clinical manifestation, with subclassifications [15] described as the following:

- Type 3a characteristics include mild visceral symptoms and the generation of myoclonic epilepsy [15,19].
- Type 3b is the most frequent subtype, distinguished by impaired saccadic eye movements and severe visceral involvement [15,19].
- Type 3c only implicates aortic calcification and the hydrocephalus and can have corneal opacities [15,19].

## 5. Physiopathology

Glucocerebrosides localize in different cell membranes, and when senescence or cellular damage occurs, macrophages may engulf them [20]. Inside macrophages, lysosomes are responsible for the breakdown and recycling of macromolecules. These require different enzymes to carry out their work. One of these is GCase, which is responsible for glucocerebrosides hydrolyzed into ceramide and glucose [7,20,21].

GBA gene variants decrease GCase activity, leading to toxic accumulation of glucocerebrosides in macrophages and promoting its transformation to Gaucher cells, which infiltrate bone marrow tissue and the spleen, liver, lungs, and brain, causing cell damage and organ dysfunction [2,8,22].

**Figure 1:**
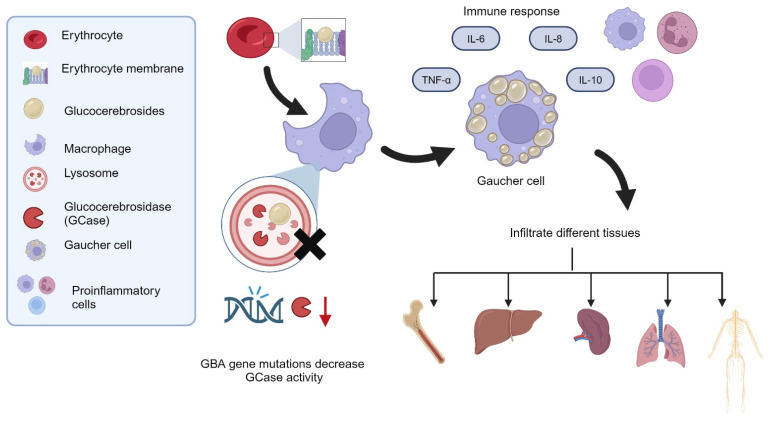
When macrophages carry out erythrocyte phagocytosis, a lysosome enzyme (GCase) in macrophages helps to hydrolyze the glucocerebrosides of the erythrocyte membrane. Reduced GCase enzymatic activity as caused by GBA gene variants results in increased glucocerebroside storage within the macrophages, thus creating the characteristic Gaucher cells that infiltrate affected tissues such as bone marrow tissue and the spleen, liver, lungs, and brain. Gaucher cells create a proinflammatory atmosphere through leukocyte chemotaxis mediated by tumor necrosis factor alpha (TNF-α) and some interleukins, such as 6, 8, and 10. This chronic proinflammatory state leads to scarring and fibrosis, which results in progressive dysfunction of the infiltrated organs and the subsequent presentation of symptoms and signs. Created with BioRender.com.

Macrophage substrate accumulation leads to an increase in inflammatory cells in the surrounding tissue and peripheral blood, such as tumor necrosis factor alpha (TNF-α) and interleukins 6, 8, and 10, in addition to macrophage inflammatory proteins 1-alpha and 1-beta (Figure 1) [2,21]. This situation leads to an immune response that is eventually submitted, such as a chronic inflammatory condition, creating scarring and fibrosis, resulting in many symptoms and signs [2,3,21].

### Physiopathology Focusing on the Nervous System

Glial cells or neuroglia are a diverse group of cells, such as astrocytes, microglia, and oligodendrocytes, that are responsible for protecting the CNS [23]. Glial cells have multiple functions, some of which are supporting immune defense, myelinization, metabolism of neurons, regulation of microenvironment compositions, formation of cerebrospinal fluid, formation of part of the blood–brain barrier and lining, repair of brain damage, and phagocytosis [24].

Microglia are cells with phagocytic properties of hematopoietic origin, and their function lies in the immune response to neuroinflammatory processes; microglial activation facilitates the release of immunosuppressive and neurotrophic factors. Once activated, microglia can initiate a proinflammatory cascade that results in the release of cytotoxic molecules, such as cytokines, complement proteins, proteases, and acute phase proteins; they also cause the chemotaxis of astrocytes around plaques and release large amounts of glutamate-inducing excitotoxicity and, consequently, cause neurodegeneration [25].

As mentioned above, variants in the human glucocerebrosidase gene are responsible for the macrophage’s transformation into Gaucher cells, which infiltrate different tissues, including brain tissue. This situation is critical in the development of neuropathic variants [26]. Mutations in the GBA gene also participate in the activation of microglia and astrocytes, induced by glucocerebrosides, which accumulate in the brain and act as a direct ligand through macrophage-inducible C-type lectin (Mincle) to induce the phagocytosis of living neurons [27]. This situation plays an essential role in the neuroinflammation associated with the disorder [28].

Recognizing this physiopathology focus in the nervous system provides a global context to understanding new types of treatment, which involve an inhibitor of microglial activation or contrasting microglial activation by deleting Mincle [27].

## 6. Diagnosis

Late or misdiagnosed GD commonly occurs due to the complex clinical presentation of this multisystemic disorder, along with a lack of knowledge about this rare disease. It can take years for the first clinical and laboratory signs to appear [8].

For the diagnostic approach of a patient with GD, it is important to take into account the types of variants and determine their nomenclature according to the Human Genome Variation Society (HGVS) guidelines for variant nomenclature. This provides important information for the clinical classification of GBA variants, which refers to the severity of GD [29]. Mild mutations cause GD type 1 and severe mutations cause GD types 2 or 3 [29]. The most prevalent pathogenic variant in patients with GD1 is the c.1226A > G, p.N409S (NM_000157.4) allele, and p.L444P is a severe variant associated with GD types 2 and 3 [29,30].

The definitive diagnosis is made by demonstrating deficient activity of the GCase enzyme and identifying characteristic mutations in the GBA gene [31]. Enzyme level measurement is determined via leukocytes or monocytes in peripheral blood or fibroblast cultures [8,22,31]. The diagnosis is confirmed when enzymatic activity results are equal to or less than 15% of normal activity [8].

Biopsies of the affected organs may identify Gaucher cells [31]. Bone marrow aspiration is not recommended for diagnosis but may help rule out other pathologies [22].

Prenatal diagnosis is only required in special situations, such as those with a family history of the disease. Genetic analysis is very useful, using a sample of chorionic villi (between 10 and 12 weeks of gestation) or amniotic fluid cells (at 16 weeks of gestation) [8]. Prenatal diagnosis can also be conducted using dried filter paper blood spots (DBSs), which is a method also used for screening [32].

Laboratory and radiological findings may also guide the disease diagnosis (Table 2) [22].

Biomarkers are an important element in assessing the severity of the disease at diagnosis, monitoring treatment effectiveness, and estimating disease progression [33]. The biomarkers that have been used frequently are ferritin, chitotriosidase, and inflammatory macrophage protein 1β. However, these biomarkers are not directly involved with the pathology of the disease, but they reflect the activation of macrophages in a secondary way, which are the target cells of the GD [33]. On the other hand, glucosylsphingosine (Lyso-Gb1) has been studied as a more specific and sensitive biomarker of GD activity and progression because this is a substrate of the deacetylated form of the GCase that accumulates in the reticuloendothelial cells, which directly reflects the insufficient amounts of the enzyme [32,33]. Lyso-Gb1 has also been studied for its function in prenatal diagnosis and DBS screening; moreover, studies also report that it can be useful in identifying the phenotype because it has been shown that plasma Lyso-Gb1 concentrations are significantly higher in GD2 and GD3 compared with GD1 [32].

### Diagnosing in Young Infants

For GD2 diagnosis, a swallowing evaluation and skin ultrastructure examination can be helpful. In these patients, a decrease in swallowing ability is common, and the gold standard is the modified barium swallow [15]; the age of onset of reported symptoms ranges from birth to 12 months, with a majority of patients exhibiting symptoms before 5 months [34]. On the other hand, the GCase enzyme regulates the ratio of ceramides to glucosylceramides in the outer layer of the skin, and lipid analyses of GD2 patients have shown that the stratum corneum has increased levels of glucosylceramide, in contrast to GD1 and GD3 patients [15].

One of the earliest signs of neurological GD is the onset of saccadic movements; for this reason, saccadic movements have begun to be evaluated through video-oculography, which is helpful for the definition of phenotypes in GD and provides patients with better care [15,35]. Usually, eye movement problems develop at 2 years post-diagnosis [35,36]. Other early neurological manifestations are the presence of seizures, failure to thrive, and developmental delay [15].

## 7. Treatment

The treatment for Gaucher disease has undergone a significant transformation since introducing enzyme replacement therapy (ERT) [21]. Previously, patients only received symptomatic treatment to alleviate the disease’s multisystemic manifestations [21,31].

Treatment goals are to eliminate symptoms, prevent complications, and improve quality of life. Due to the heterogeneous nature of the disease [31], treatment should be individualized. Currently, two specific types of treatment available are the following: enzyme replacement therapy (ERT) (Table 3) and substrate reduction therapy (SRT) [8,37].

### 7.1. Enzyme Replacement Therapy (ERT)

Enzyme replacement therapy (ERT) is effective for type 1 Gaucher disease, as it improves most clinical manifestations [22]. Moreover, it can also benefit patients with type 3 Gaucher disease with chronic visceral manifestations. However, ERT is not recommended for patients with type 2 Gaucher disease, as it does not halt disease progression [39].

ERT aims to provide a recombinant GCase enzyme targeted at macrophages, thereby replacing deficient enzymatic activity and enabling the degradation of glucocerebrosides [8].

The Food and Drug Administration (FDA) has approved Cerezyme (imiglucerase), produced from mammalian cells (Chinese hamster ovary cells), and VPRIV (velaglucerase alfa), produced from human fibroblasts, for the management of Gaucher disease types 1 and 3 [8,22,40]. Elelyso (taliglucerase alfa) differs from the above drug treatments because recombinant DNA technology is used in plants and is produced in carrot cells transfected with the human β-glucocerebrosidase gene; Elelyso is used to treat GD type 1 [41]. Tama Dinur et al.’s 2021 study cohort showed that Lyso-Gb1 levels were lower for patients treated with velaglucerase alfa and taliglucerase alfa than for patients treated with imiglucerase [38]. ERT is ineffective in treating central nervous system problems associated with types 2 and 3 of the disease [40].

The initial treatment dose is determined individually based on the severity and clinical progression of the disease [21]. Most patients start with 30–60 U/kg doses every two weeks, with a long-term maintenance dose of 30 U/kg every two weeks [21,22].

The expected effects of ERT include an increase in hemoglobin levels and platelet count, a reduction in hepatosplenomegaly, and a decrease in angiotensin-converting enzyme and acid phosphatase levels [8,39]. In pediatric patients, growth delay improves [39]. Bone marrow infiltration and osteopenia gradually regress [8].

If the patient does not respond to the treatment after 6 months, doses are increased until the desired results are achieved [21]. Furthermore, ERT is a lifelong treatment, as discontinuation is associated with relapses [21,22].

### 7.2. Substrate Reduction Therapy (SRT)

Substrate reduction therapy (SRT) aims to reduce toxic levels of glucocerebrosides by inhibiting glucosylceramide synthase (GCS), the enzyme responsible for the biosynthesis of glucocerebrosides [42,43]. Additionally, the therapy allows the small amount of substrate that is still produced to be degraded by the residual GCase in all patients with type 1 Gaucher disease [43].

There are two FDA-approved medications for this therapy: miglustat and eliglustat [42]. Miglustat is a non-specific, reversible inhibitor of GCS. It is a second-line treatment indicated for patients with GD type 1 who no longer tolerate ERT or who have developed intolerance to the therapy [42,43]. Although this drug can cross the blood–brain barrier, it does not affect neurological symptoms [8]. Eliglustat, a ceramide analog, is a reversible, potent, and selective inhibitor of GCS [43]. It is approved for treatment-naïve adults and patients previously treated with ERT. The dosage of 84 mg once or twice daily depends on the cytochrome P450 2D6 genotype (extensive, intermediate, or poor metabolizer) [44]. Eliglustat is a first-line treatment for adults with GD type and is more specific and potent than miglustat [8,42]. However, Torralba-Cabeza [43] reports that “there is no significant difference between the two drugs in terms of increasing blood hemoglobin, platelet count, and reducing liver and spleen size” [43].

### 7.3. Other Types of Treatments

Clinical trials are currently underway for Gaucher disease, searching for alternative therapies, including gene therapy, small molecule glucocerebrosidase chaperones, microglia-mediated treatment, ambroxol, isofagomine, or nanovesicles [33].

### 7.4. Pharmacological Treatments

#### 7.4.1. Gene Therapy

Gene therapy aims to modulate gene expression to achieve a therapeutic effect. It has the following two modalities:

- Ex vivo: This therapy involves extracting the patient’s cells, transducing them with a lentiviral gene (a virus with a prolonged incubation period), and returning them to the patient once they are conditioned [45,46].
- In vivo: In this therapy, vectors such as adeno-associated viruses are used to reach the target [45,46].

Gene therapy allows the introduction of healthy copies of a gene to replace defective ones to interrupt the function of mutated genes or to add a new gene with therapeutic effects. This gene therapy can help the GBA gene function properly to produce GCase [46]. The use of GBA gene vectors in hematopoietic stem cells, liver cells, and even brain cells is under study [47]. Since GD can have neurological involvement, this treatment has the advantage that many vectors can cross the blood–brain barrier [46]. One of the most commonly used vectors is adeno-associated viruses due to their neurotropic properties, which allow them to cross the blood–brain barrier and integrate the therapeutic gene into damaged cells, enabling proper GCase synthesis [48].

#### 7.4.2. Pharmacological Chaperones

Pharmacological chaperones are low-molecular-weight compounds designed to bind exclusively to a target protein to facilitate its folding and stimulate lysosomal translocation [49]. These compounds include competitive and reversible active site inhibitors that serve as binding agents for GCase, reducing enzyme retention in the endoplasmic reticulum and preventing its degradation, thus facilitating translocation to the lysosome [47]. This therapy can increase the enzyme’s stability and catalytic activity, allowing the cell to function normally by removing accumulated compounds [49]. The first chaperone studied for GD was N-octyl-b-valienamine, which improved the activity of the mutant F2131 GCase [48].

#### 7.4.3. Microglia-Mediated Treatment

Microglia are a new, promising therapeutic target, especially for neuropathic variants [50]. In Shimizu et al.’s 2023 study, postmortem brains of neuropathic GD patients and control donors were analyzed. The authors found activated microglia with phagocytic markers expressed and phagocytosing living neurons in the GD cortex. TNF expression by microglia was also detected in patients with GD [27]. The study results led the authors to search for therapeutic options by targeting this shared pathway. They found that minocycline and etanercept effectively decreased microglial activation, protected neurons, diminished neurological phenotypes, and prevented early death [27,50].

#### 7.4.4. Ambroxol

Ambroxol is another drug that has been studied for this disease, and it may work in patients with GD by stabilizing the misfolded GCase enzyme in the endoplasmic reticulum and preventing its recognition by the cell. Ambroxol then dissociates from the enzyme, allowing it to break down accumulated substrates [49]. Other mechanisms studied include reducing the concentration of hexosylsphingosine, enhancing the activity of mutant β-glucosidase, and stimulating the unfolded protein response [51]. Ambroxol is classified as a safe drug with mild side effects, most of which are gastrointestinal, such as nausea, vomiting, or discomfort [49]. It has also shown optimal results in some patients with neurological symptoms, such as controlling seizures and gait [52]. One limitation of ambroxol is that it acts as an inhibitory chaperone, unlike other compounds that function as inhibitors while enhancing their target’s effect [53].

#### 7.4.5. Isofagomine

Isofagomine is a substrate-mimetic compound based on iminosugars [53]. Recent studies have shown that it can increase GCase enzymatic activity in vitro in fibroblasts from patients. Isofagomine is an up-and-coming therapeutic option due to its specificity in binding to the enzyme. More studies are required to understand this compound further and optimize its function [52]. Moreover, isofagomine should be used at sub-inhibitory concentrations, and its therapeutic role is both as a chaperone and an inhibitor, although it has poor selectivity against related hydrolases [53].

#### 7.4.6. Nanovesicles

A different approach is directly administering ERT into the central nervous system using nanovesicles that can cross the blood–brain barrier [52]. These vesicles, known as SapC-DOPS-GCase, penetrate the barrier via phosphatidylserine, which is found in blood vessels, neurons, astrocytes, and microglia [52]. When administered, functional GCase is transported by these vesicles, preserving its function. To date, animal studies have shown good neurological results [52].

#### 7.4.7. Skeletal Treatment

Specific treatments for the disease, such as ERT and SRT, can alleviate skeletal manifestations. However, reversing secondary bone changes is more challenging, making it essential for patients to achieve optimal bone density to prevent pathological fractures [54]. In addition to specific therapies, calcium and vitamin D supplementation should be included to support bone mineral health [55]. Splenectomy is an important treatment in disease progression. However, it presents an additional risk factor for osteonecrosis because, following splenectomy, an overload of Gaucher cells in the bone marrow can lead to alterations in thrombosis, thrombolysis, platelet activation, and other complications [55]. Eliglustat, which was previously mentioned, has shown therapeutic benefits, including the restoration of bone mineral density to a healthy reference range, reduction in disease burden in the bone marrow, decreased bone pain, and reduced incidence of fractures [54].

### 7.5. Non-Pharmacological Treatments

Bone marrow transplantation involves transplanting hematopoietic stem cells from a healthy, compatible donor directly into the patient’s bone marrow. The transplanted stem cells generate new, healthy blood cells that can break down accumulated waste in the patient’s body [48], offering the potential for a cure; however, no clinical trials have evaluated its safety and efficacy compared with enzyme replacement therapy or substrate reduction therapy [22].

The literature has also reviewed the surgical management of Gaucher disease, including splenectomy. However, it is recommended to avoid splenectomy, which should only be performed under exceptional circumstances, such as when there is no response to treatment, persistent severe cytopenia, or in cases of splenic rupture [8,22].

## 8. Prognosis

The quality of life for patients with Gaucher disease has significantly improved in recent years. Implementing appropriate treatment with optimal therapeutic doses allows for the reversal of cytopenia and organomegaly and a significant reduction in bone manifestations [8].

However, there are cases where the outcomes may be unfavorable due to aggressive bone disease, the development of Parkinson’s disease, or a hematologic malignancy [8].

The literature describes a correlation between residual enzymatic activity and patient prognosis at diagnosis, known as the Protein Severity Index. This index reflects the severity of the genotype present in patients [56].

## 9. Conclusions

Gaucher disease is a rare but treatable metabolic disorder characterized by multisystemic clinical presentation with phenotypic variability ranging from very mild to severe and life-threatening forms. Due to its diverse and gradual clinical onset, diagnosis of GD has become a challenge for the healthcare system, requiring high suspicion for confirmation, with most cases identified after irreversible complications have developed. Early treatment is beneficial in halting or reversing the progression of the disease. ERT is very effective for most patients with type 1 GD and helps manage non-neurological complications in type 3.

Regarding advances in oral therapy, such as SRT, it could replace intravenous ERT due to cost considerations, ease of use, and the fact that patients may eventually become fatigued and resistant to venipuncture.

There is ongoing research into the efficacy of ERT followed by hematopoietic stem cell transplantation as an alternative treatment for patients with type 3 Gaucher disease who are at high risk of fatal neurological progression.

Clinical trials are underway to investigate new treatments for GD. Advances in research are essential to improve the quality of life for patients and, in many cases, offer new therapeutic options. Current treatments, such as ERT and SRT, have been effective for some patients, but limitations still exist, such as accessibility, long-term efficacy, and side effects. Therefore, clinical trials for new therapies could be crucial for finding more effective, less invasive, and more accessible treatments. New forms of treatment have been studied, mainly focusing on neuropathic variants, which offer promising therapies for patients who normally have a poor prognosis.

The success of Gaucher disease treatment not only improves patients’ quality of life but also prevents or reduces the risk of late-onset complications associated with the disease, such as the development of Parkinson’s disease and cancer, particularly plasma cell neoplasms and other hematologic malignancies.

## Figures and Tables

**Table 2:** Laboratory and imaging findings in Gaucher disease.

Laboratory Findings	Imaging Findings
Anemia Thrombocytopenia Leukopenia ↑ Liver enzyme levels Poly- and monoclonal gammopathy Lipid-laden macrophages in tissues (bone marrow, liver, and spleen) ↓ GCase activity Biomarkers ↑ Ferritin ↑ Chitotriosidase ↑ Serum angiotensin-converting enzyme (ACE) ↑ Inflammatory macrophage protein 1β ↑ Lyso-Gb1	Bone X-ray ○ Erlenmeyer flask deformity ○ Bone fractures and lytic lesions Chest X-ray ○ Pulmonary infiltrate Magnetic resonance imaging (MRI) ○ Bone marrow involvement ○ Bone infarctions ○ Osteonecrosis Dual-energy X-ray absorptiometry (DEXA) ○ Osteopenia Abdominal ultrasound ○ Hepatomegaly ○ Splenomegaly Echocardiography Pulmonary hypertension

Modified from [22]. With actualizations from [32,33]. ↑: Increase, ↓:decrease.

**Table 3:** Criteria for initiating ERT.

I. Confirmed diagnosis of Gaucher disease by determining GCase enzyme levels in plasma, leukocytes, tissue biopsy, or fibroblast culture.
II. The presence of one or more of the following manifestations: In at least two measurements taken one month apart, hemoglobin decreased 2.0 g/dL below the normal limit for age and sex, with other causes of anemia excluded. A platelet count below 100,000/mm^3^ in at least two measurements taken one month apart. Liver size ≥ 1.25 times the normal size (as determined using computed tomography). Spleen size ≥ 10 times the normal size (as determined using computed tomography). Previous splenectomy. Bone disease evidenced by any abnormalities: avascular necrosis, lytic disease, pathological fracture, failure of bone remodeling, bone marrow infiltration, osteopenia, or osteosclerosis. Pulmonary involvement. Children with a history of siblings with severe or progressive disease. Children with growth retardation in weight and height over a 6–12-month period that is not attributable to other causes. Molecular evidence of the L444P mutation in the homozygous state.
III. Neurological abnormalities associated with type 2 neuropathic Gaucher disease.
IV. An increase in plasma Lyso-Gb1 (>250 ng/mL).

Modified from [37]. With actualizations from [30,38].

## Data Availability

No new data were created or analyzed in this study. Data sharing is not applicable to this article.
